# An Experiencer, An Animal or An Object? Erection Salience Decreases Men’s Perceived Agency

**DOI:** 10.1007/s10508-020-01800-0

**Published:** 2020-09-07

**Authors:** Paulina Górska, Magdalena Budziszewska, Marta Marchlewska, Anna Stefaniak, Katarzyna Malinowska, Olga Kuzawińska

**Affiliations:** 1 Faculty of Psychology, University of Warsaw, ul. Stawki 5/7, 00-183 Warsaw, Poland; 2 Institute of Psychology, Polish Academy of Sciences, Warsaw, Poland; 3 Department of Psychology, Carleton University, Ottawa, ON Canada

**Keywords:** Mind perception, Penile erection, Penis size, Objectification, Dehumanization

## Abstract

Three experiments investigated the influence of penile erection on ascriptions of mental capabilities to men. Drawing on sexual objectification literature and the distinction between agency and experience in mind perception, three competing predictions were formulated. The mind redistribution hypothesis assumed that penile erection would lower agency and heighten experience attributions, the animalistic dehumanization hypothesis predicted the decrease in agency, but not experience, and the literal objectification hypothesis implied the simultaneous decrease in both agency and experience. In Experiment 1 (*N *= 219; 128 females), erection salience lowered agency, but not experience capabilities ascribed to male targets. Experiment 2 (*N *= 201, 113 females) replicated the negative effect of erection salience on perceived agency (but not experience) and revealed that erection salience lowered intentions to hire a male target. This effect was explained with the loss of perceived agency. Experiment 3 (*N *= 203, 98 females) verified the causal relationship between penile erection, agency and hiring intentions. Taken together, these results supported the animalistic dehumanization hypothesis.

**Electronic supplementary material:**

The online version of this article (10.1007/s10508-020-01800-0) contains supplementary material, which is available to authorized users.

## Introduction

The association between men’s penises and their assumed mental capacities can be found in numerous cultures (Friedman, 2001). For instance, men’s mental facilities have been linked to the size of their genitals. In Ancient Greek art, representations of barbarians abounded with large penises, which stood in stark contrast with the small phalluses of civilized Greeks (Friedman, 2001). On the other hand, an erect (vs. flaccid) penis is believed to override higher thought processes (Hickman, 2012). St Augustine of Hippo (397–426/1996) claimed that men’s sexual arousal suspends their mental activity. The same idea has inhabited folk wisdom: “When the prick stands up, the brain gets buried in the ground,” said the Yiddish proverb (Roth, 1969). In fact, this part of common knowledge receives support from results of scientific research showing that sexual arousal impairs higher-order neurocognitive processes (e.g., Macapagal, Janssen, Fridberg, Finn, & Heiman, 2011; Suchy et al., 2019).

From a psychological perspective, the trade-off between penis properties and the perceived mind of its possessor may be explained in terms of sexual objectification—reducing a person to sexual body parts or functions (Gervais, Bernard, Klein, & Allen, 2013). Research shows that sexualized targets are attributed lesser mental capacities than their non-sexualized counterparts (e.g., Daniels & Zurbriggen, 2016). This effect occurs because of the shift in the perceiver’s attention from a given person’s intellect to their physical aspects. Although sexual objectification has been demonstrated predominantly for female targets (see Heflick & Goldenberg, 2014), some studies suggest that men may also be objectified (e.g., Fasoli, Durante, Mari, Zogmaister, & Volpato, 2017; Loughnan et al., 2010).

Increasing prevalence of sexualized images of men (alongside women) in the mass media (Flynn, Park, Morin, & Stana, 2015; Hatton & Trautner, 2011) begs a question of how such imagery might impact perceptions of sexualized male targets. While there may be different levels of sexualization—from presenting non-sexualized targets, through targets that are scantily dressed or nude, to nude targets in sexual poses (Fasoli et al., 2017)—in the current paper we focus on the influence of the salience of male genitals, either through erection or size and its influence on the quantity of mind attributed to a man. Due to limited research on sexual objectification of men, we were interested in verifying which feature of the presented male genitalia (size vs. erection) would have a greater influence on perception of a male’s mind. Previous studies established that more revealing portrayals, and especially those that were both nude and sexual, were associated with changes in ascription of mind to the targets (Fasoli et al., 2017; Gray, Knobe, Sheskin, Bloom, & Barrett, 2011b). Therefore, because erection salience, and not simply the size of a penis, is a more direct indicator of a man’s sexual readiness, we expected that it would affect mind perception to a greater extent than penis size. This raises an intriguing question: Which exact aspects of the male mind will be differently perceived by respondents vis-à-vis erect versus flaccid penis conditions?

Mind was traditionally understood as a unidimensional phenomenon that beings possess to different extents. Gray, Gray, and Wegner (2007) distinguished two dimensions of mind: agency and experience. While agency refers to the capacity to act, plan and exert self-control, experience denotes the capability to feel pain, pleasure and emotions. These two dimensions translate to moral intuitions (Gray et al., 2011b) and depend on target and perceiver characteristics (Gray et al., 2007; Gray, Jenkins, Heberlein, & Wegner, 2011a).

Consequences of objectification that primarily denies the experiential versus agentic aspects of the human mind differ. Denying experience leads to less care for the objectified other and increases likelihood of aggression and violence toward them, including acceptability of rape, pain infliction and sexual harassment (Heimerdinger-Edwards, Vogel, & Hammer, 2011; Loughnan, Pina, Vasquez, & Puvia, 2013). In broader terms, it relates to denial of rights (Gray et al., 2007). Denying agency, on the other hand, has consequences for the status afforded to a person in society. It plays an especially important role in work environments, influencing hiring or promotion decisions (Loughnan & Pacilli, 2014). In a more general sense, it effects the perception of a person as a moral agent, capable of taking and exercising responsibility (Gray et al., 2007).

Given the novel topic of our research, we formulated three divergent predictions with regard to the influence of the target’s erection on ascription of agency and experience. First, high erection salience may diminish agency and increase experience (H1). According to the redistribution of mind hypothesis (Gray et al., 2011b), a focus on the body results in the reallocation of agency and experience capacities ascribed to a person: When viewed as their bodies, people are seen as experiencers, who may feel pain and emotions, but lack in agency-related capabilities. Thus, a body focus does not lead to dementalization (i.e., the total amount of perceived mind remains unaltered), but it changes the relative importance of agency and experience in favor of the latter. In a series of studies comparing naked to clothed and to sexually suggestive targets, the latter were ascribed the most experience and the least agency (Gray et al., 2011b).

Alternatively, a visible erection may lower the perception of the target’s agency and have no effect on experience (H2). This implies that an erection would lead to some degree of dementalization, as the total amount of perceived mind is reduced. The loss of agency, but not experience, corresponds to the animalistic type of dehumanization (Haslam, 2006): The target is denied uniquely human traits such as moral sensibility, but still attributed qualities that reflect human nature, such as emotional responsiveness (see Gray et al., 2007). Indeed, in comparison with personalized targets, sexually objectified individuals have been shown to induce stronger associations with animals (Vaes, Paladino, & Puvia, 2011, Study 1) and to receive lower ratings of agency, but not experience (Holland & Haslam, 2013).

Finally, erection salience may decrease ratings of target’s agency and experience (H3). This would be tantamount to literal objectification—perceiving a person as object-like and denying their subjectivity (Bernard, Gervais, Allen, Campomizzi, & Klein, 2012; Heflick & Goldenberg, 2014; Nussbaum, 1995). Seeing others as objects entails the largest degree of dementalization and may have especially dire consequences (e.g., Loughnan et al., 2013). Similar to the other two possibilities, the denial of agency- and experience-related capacities to sexualized targets has been found in previous studies (e.g., Heflick & Goldenberg, 2009).

The present research aimed to test these three competing predictions against each other and to explore the most promising one. To this end, we carried out three experiments. The objective of Experiment 1 was to explore whether and how the features of a penis influence agency and experience ascribed to male targets. Experiment 2 was intended to check whether the changes in mind perception due to erection salience translate into real-life consequences such as hiring decisions. Finally, Experiment 3 aimed at establishing the causal relationship between mind ascription and hiring decisions.

## Experiment 1

One context in which presenting male genitals is acceptable is art. Capitalizing on this circumstance, we selected three male nude drawings on the Internet and then altered them to differ in size and erection salience.^1^ The manipulated images served as the stimuli presented to participants.

## Method

### Participants

A sample of *N *= 219 British residents aged 19–69 years (128 females and 91 males; *M*_age_ = 38.75, SD_age_ = 13.25) recruited via Prolific Academic (Peer, Brandimarte, Samat, & Acquisti, 2017) participated in the study online. Sample size was predetermined to be at least 50 individuals per experimental condition (Simmons, Nelson, & Simonsohn, 2013). The final number of participants yielded 100% power to detect a medium-size (*ƒ* = .25) within–between interaction, assuming a correlation of .60 between the ratings for agency and experience (e.g., Hughes & Trafimov, 2015). No observations were excluded. Informed consent was obtained from all individual participants.

## Materials and Procedure

**Fig. 1:**
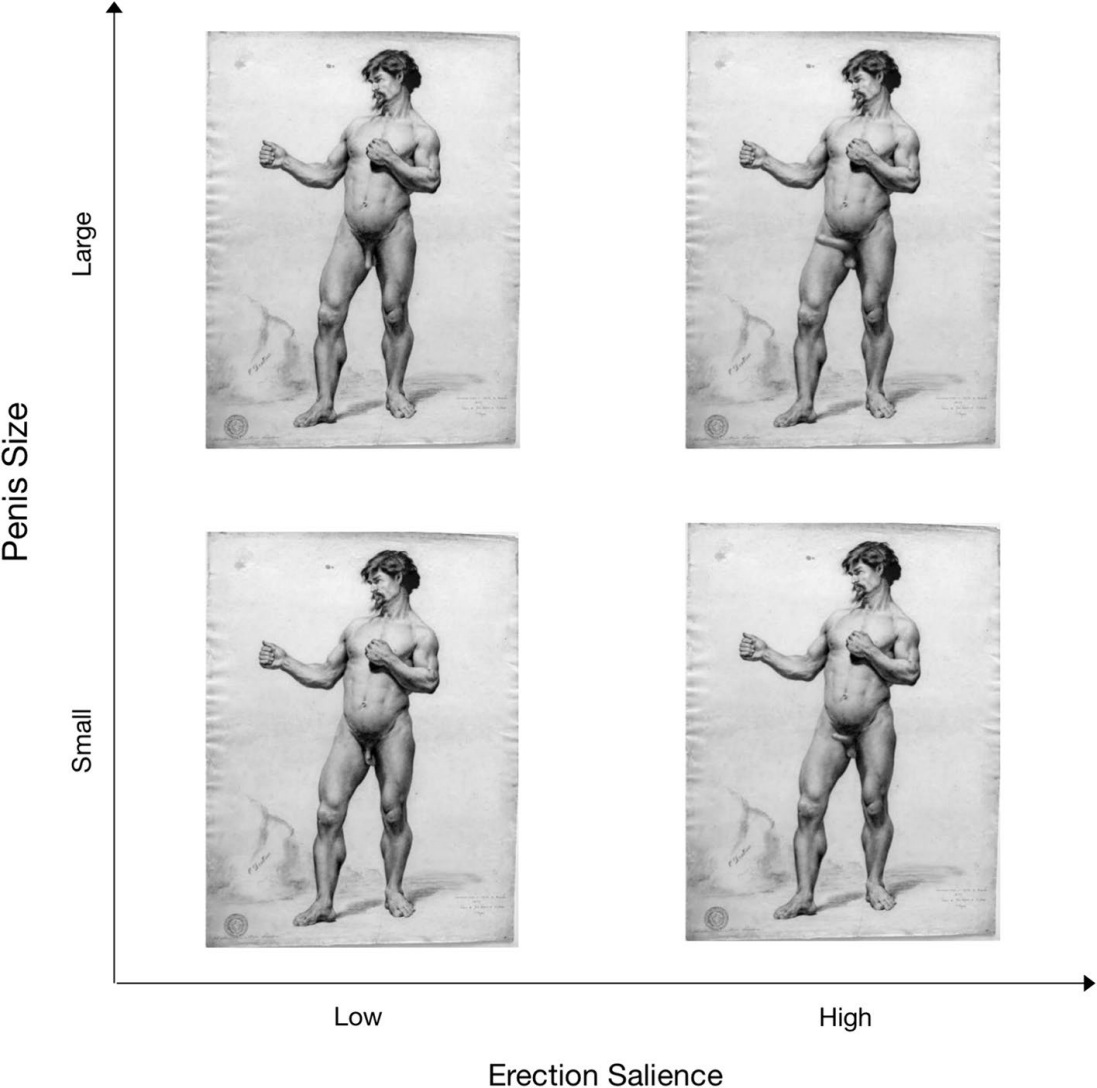
Sample stimuli used in Experiment 1

Stimuli were three male nude drawings that manipulated (in a 2 × 2 design) whether the targets’ penises were small or large and whether they were erect or not.^2^ Figure 1 shows the four versions of a sample drawing. The drawings were made in convention typical for fine arts, and the background was kept gray to avoid interferences from the context.

After random assignment to one of the four experimental conditions, participants were asked to evaluate three drawings presented in a random order.^3^ To assess the perception of targets’ mental capacities, we used the scale provided by Gray et al. (2011b). For each of the three drawings, participants were asked to evaluate to what extent the man in the picture was capable of exhibiting six mental capacities (1 = *not at all capable*, 7 = *fully capable*). While three items (exercising self-control, remembering and acting morally) tapped into agency-related properties, the other three (feeling fear, pleasure and hunger) assessed experience-related capacities. The responses to specific items were averaged across pictures and then used to form composite scores for (averaged) agency (*α* = .87, *M* = 4.96, SD = 1.12) and experience (*α* = .76, *M* = 5.37, SD = 1.02).^4^ There was a positive correlation between agency and experience ratings, *r*(217) = .74, *p* < .001.

## Results

### Agency and Experience Ratings

**Fig. 2:**
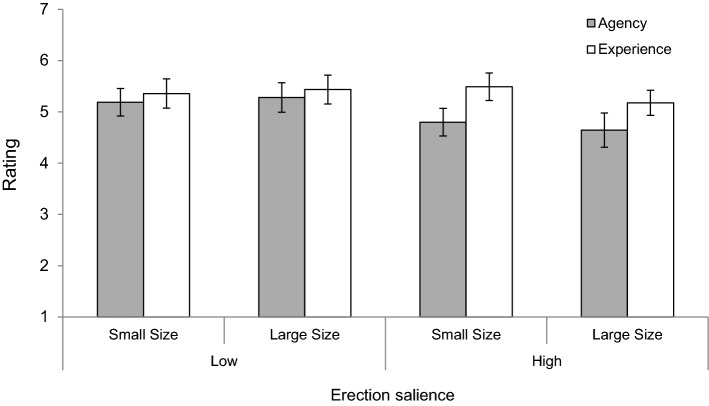
Results of Experiment 1: Mean ratings of agency and experience as a function of target’s penis size and erection salience. Error bars show 95% confidence intervals

To analyze the data, we used a 2 (Penis Size: Small vs. Large) × 2 (Erection Salience: Low vs. High) × 2 (Mental Capacity: Agency vs. Experience) mixed-design ANOVA. While penis size and erection salience served as between-subjects factors, mental capacity was treated as a within-subjects factor. To adjust for multiple comparisons (in this study as well as in the remaining ones), we employed a Bonferroni correction. Figure 2 presents the mean ratings of agency and experience across all experimental conditions.

The main effect of penis size was not significant, *F*(1, 215) = 0.31, *p* > .250, *η*_*p*_^2^ = .00, while the main effects of erection salience, *F*(1, 215) = 4.56, *p* = .034, *η*_*p*_^2^ = .02 and mental capacity, *F*(1, 215) = 57.31, *p* < .001, *η*_*p*_^2^ = .21, were significant. The main effects of erection salience and mental capacity were qualified by a two-way interaction, *F*(1, 215) = 19.30, *p* < .001, *η*_*p*_^2^ = .08. Probing this interaction revealed that targets with a visible erection were ascribed lower agency (*M* = 4.73, SD = 1.17) than targets with no erection (*M* = 5.23, SD = 1.00), *F*(1, 215) = 11.78, *p* = .001, *η*_*p*_^2^ = .05. By contrast, targets in the low (*M* = 5.40, SD = 1.03)- and the high-erection-salience condition (*M* = 5.35, SD = 1.02) did not differ in terms of perceived experience, *F*(1, 215) = 0.20, *p* > .250, *η*_*p*_^2^ = .00. The difference between experience and agency ratings was larger in the high, *F*(1, 215) = 77.29, *p* < .001, *η*_*p*_^2^ = .26, than the low-erection-salience condition, *F*(1, 215) = 4.70, *p* = .031, *η*_*p*_^2^ = .02. No other interactions were significant, *p*s > .250. Consistent with past results (e.g., Holland & Haslam, 2013), further analyses showed no significant main or interaction effects for participants’ gender when it was included as an additional factor, *p*s > .108. (These analysis are presented in Supplemental Material.) Similarly, excluding non-heterosexual participants (*n* = 20; see Fasoli et al., 2017) or including a variable for the drawings as an additional within-subjects factor did not affect the results in a meaningful way.

## Experiment 2

Supporting H2, erection salience (regardless of penis size) lowered perceived agency, but not experience of male models. In Experiment 2, we aimed to replicate this finding in a different cultural setting. Past research showed that objectification may decrease men’s perceived suitability for certain professions (Rollero & Tartaglia, 2013). However, the exact mechanism behind this effect is unclear. As agency-related traits are desirable on the job market (e.g., Dunn, Mount, Barrick, & Ones, 1995), we hypothesized that the decrease in perceived agency due to high erection salience would translate into lower willingness to hire a target. To ensure that this effect was not occupation-specific, we measured hiring intentions in relation to two professions—a nurse and a security guard—that differ in terms of prestige (e.g., Smith & Son, 2014) and compatibility with a masculine gender role (Eagly, Wood, & Diekman, 2000).

Since body focus may affect perception differently depending on the level of the target’s sexualization (Fasoli et al., 2017), less explicit stimuli were used. Experiment 2 also accounted for some limitations of Experiment 1. In particular, given the superior psychometric properties of multi-item measures (Judd, Smith, & Kidder, 1991) we employed a more comprehensive measure of mind perception (Gray et al., 2007) as well as a manipulation check for erection salience. To reflect the shift to the confirmatory phase of the present research (Van’t Veer & Giner-Sorolla, 2016), Experiment 2 was preregistered prior to data collection (https://osf.io/gkcb5).

## Method

### Participants

Two hundred and one^5^ users of a university library in Warsaw, Poland, aged 19–47 years (113 females, 85 males and 3 undeclared; *M*_age_ = 23.13, SD_age_ = 3.77; 87.6% university students) took part in the study. Based on 80% power, correlation between the repeated measures (*r* = .74, *p* < .001) and the effect size (*η*_*p*_^2^ = .08) obtained in Experiment 1, 96 participants would be needed to detect an erection salience × mental capacity interaction effect in the present study. However, because we deemed the present manipulation to be less powerful than the pictures employed in Experiment 1, the sample size was predetermined to 200, which would enable the detection of even small effects (*ƒ *= .07 assuming 80% power). No observations were excluded. Informed consent was obtained from all participants.

### Materials and Procedure

**Fig. 3:**
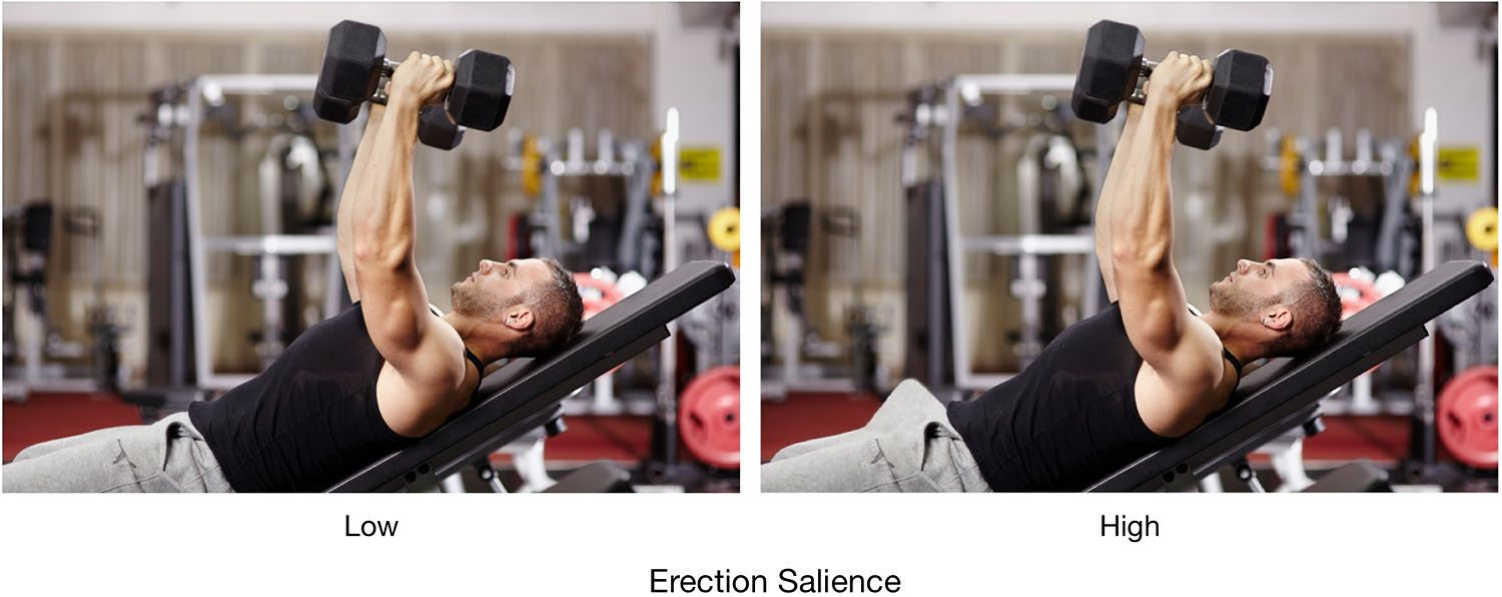
Stimuli used in Experiment 2

Participants were recruited in a university library building. They were approached by research assistants and invited to take part in a study introduced as research on the hiring process. If they consented to participate, they were led to a self-study room where they completed a questionnaire. In this experiment, the participants were asked to take a role of a human resources manager who inspects the social media profiles of job applicants to inform their decision-making. Following random assignment to one of the two experimental conditions (erection salience: low vs. high), participants were presented with a photograph of a man working out in a gym. Tomasz, the job applicant in the picture, was allegedly photographed by a colleague, who then published and tagged the photograph online. The photographs differed between the conditions (see Fig. 3). Next, mind perception and hiring intentions were assessed in a counterbalanced order. First, participants were asked to rate to what extent Tomasz was capable of displaying 10 mental capacities, a measure previously used by Gray et al. (2007) (1 = *not at all capable*, 7 = *fully capable*). The composite score for agency (*α* = .87, *M* = 4.70, SD = 1.29) was created by averaging responses to five items: exercising self-control, acting morally, planning, communicating and recognizing emotions of others. Similarly, averaging responses to the remaining five items (feeling hunger, pleasure, desire, pain and rage) produced the composite score for experience (*α* = .81, *M* = 5.31, SD = 1.12). Agency and experience correlated positively, *r*(199) = .58, *p* < .001.

Intentions to hire the target as a security guard (*α* = .77, *M* = 5.13, SD = 1.30) and a nurse (*α* = .86, *M* = 3.52, SD = 1.39) were assessed with three items each (Hansen & Dovidio, 2016): “I would hire Tomasz for the position of a security guard/nurse,” “Tomasz is qualified for the position of a security guard/nurse,” “Tomasz is a good fit for the position of a security guard/nurse” (1 = *definitely not*; 7 = *definitely yes*). The composite scores were created by averaging security guard- and nurse-related items and correlated positively, *r*(198) = .33, *p* < .001.

Mind perception and hiring intentions scales were followed by a manipulation check. To verify whether the erection salience manipulation was successful, participants expressed their agreement or disagreement with the following statement: “Tomasz had an erection” (1 = *definitely not*, 7 = *definitely yes*), *M* = 4.00, SD = 2.12.

## Results

### Manipulation Check: Erection Ratings

In comparison with the low-erection-salience condition (*M* = 2.72, SD = 1.42), participants assigned to the high-erection-salience group (*M* = 5.23, SD = 1.95) were more strongly convinced that Tomasz had an erection, *F*(1, 196) = 105.88, *p* < .001, *η*_*p*_^2^ = .35.

### Agency and Experience Ratings

**Fig. 4:**
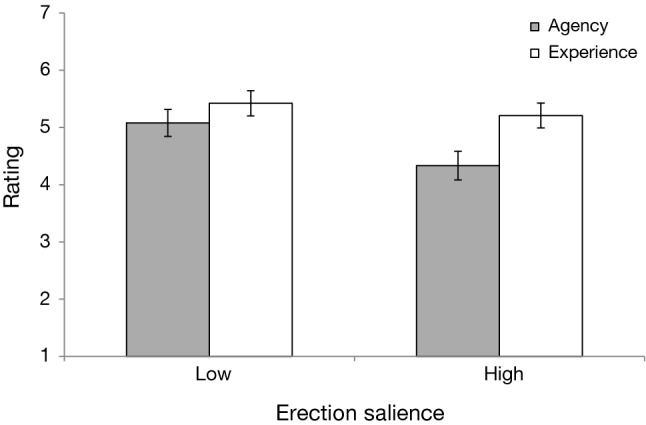
Results of Experiment 2: Mean ratings of agency and experience as a function of target’s erection salience. Error bars show 95% confidence intervals

A 2 (Erection Salience: Low vs. High) × 2 (Mental Capacity: Agency vs. Experience) mixed-design ANOVA revealed significant main effects of mental capacity, *F*(1, 199) = 62.22, *p* < .001, *η*_*p*_^2^ = .24 and erection salience, *F*(1, 199) = 10.61, *p* = .001, *η*_*p*_^2^ = .05. These effects were qualified by a two-way interaction of erection salience and mental capacity, *F*(1, 199) = 12.05, *p* < .001, *η*_*p*_^2^ = .06. Participants in the high-erection-salience condition evaluated the target as less agentic (*M* = 4.33, SD = 1.29) than subjects in the low-erection-salience condition (*M* = 5.08, SD = 1.19), *F*(1, 199) = 18.22, *p* < .001, *η*_*p*_^2^ = .08. On the other hand, mean ratings of experience did not differ between low (*M* = 5.42, SD = 1.11)- and high-erection-salience conditions (*M* = 5.21, SD = 1.11), *F*(1, 199) = 1.84, *p* = .177, *η*_*p*_^2^ = .01. Ratings of the target’s experience and agency differed more strongly in the high, *F*(1, 199) = 65.48, *p* < .001, *η*_*p*_^2^ = .25, as compared to the low-erection-salience group, *F*(1, 199) = 9.61, *p* = .002, *η*_*p*_^2^ = .05. Participants’ gender did not qualify the erection salience and mind perception interaction, *F*(1, 194) = 1.96, *p* = .163, *η*_*p*_^2^ = .01 (see Supplemental Material). Mean ratings of agency and experience are shown in Fig. 4.

### Hiring Intentions

Next, we performed a 2 (Erection Salience: Low vs. High) × 2 (Profession: Security guard vs. Nurse) mixed-design ANOVA to check whether erection salience changed participants’ willingness to hire the target as a security guard and a nurse. There was a main effect of erection salience, *F*(1, 198) = 13.32, *p* < .001, *η*_*p*_^2^ = .06, with lower overall hiring intentions in the high (*M* = 4.06, SD = 1.20)- as compared to the low-erection-salience condition (*M* = 4.61, SD = 0.91). The main effect of profession was also significant, *F*(1, 198) = 211.14, *p* < .001, *η*_*p*_^2^ = .52. In line with our expectations, participants reported higher intentions to employ the target as a security guard (*M* = 5.13, SD = 1.30) than a nurse (*M* = 3.52, SD = 1.39). Importantly, a two-way interaction between erection salience and profession did not reach significance, *F*(1, 198) = 1.07, *p* > .250, *η*_*p*_^2^ = .01, suggesting that the effect of erection salience did not depend on the type of job. Adding gender as a between-subjects factor did not change this pattern of results (see Supplemental Material).

### Mediation of the Erection Salience–Hiring Intentions Link by Agency and Experience

**Fig. 5:**
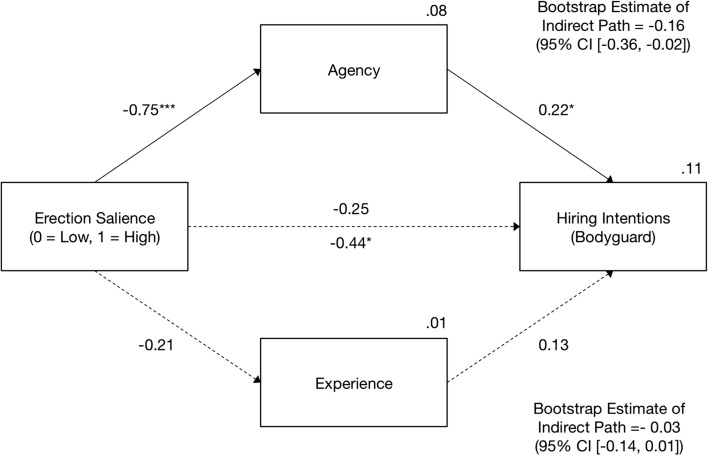
Results of the mediation analysis in Experiment 2: The effect of erection salience on intentions to hire the target as a security guard. On the path from erection salience to hiring intentions, the direct effect is shown above and the total effect is shown below the arrow. The entries are unstandardized effects. Solid arrows show significant effects (*** *p* < .001, * *p* < .05). CI = bootstrap bias-corrected confidence intervals

**Fig. 6:**
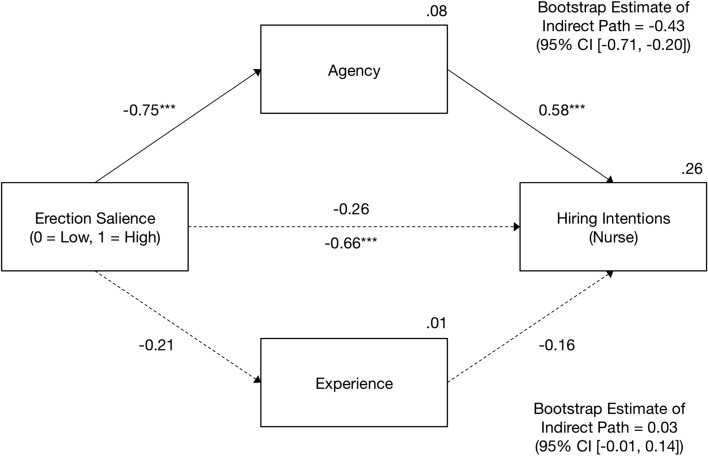
Results of the mediation analysis in Experiment 2: The effect of erection salience on intentions to hire the target as a nurse. On the path from erection salience to hiring intentions, the direct effect is shown above and the total effect is shown below the arrow. The entries are unstandardized effects. Solid arrows show significant effects (*** *p* < .001). CI = bootstrap bias-corrected confidence intervals

We tested two mediation models to check whether the decrease in hiring intentions due to high erection salience could be explained with changes in mind perception. While erection salience served as the focal predictor, perceived agency and experience were treated as parallel mediators and intentions to hire the target as a security guard and a nurse constituted two respective dependent variables. Two mediation models were estimated with Model 4 of PROCESS macro (Hayes, 2013) using a bootstrap procedure with 5000 resamples. As shown in Figs. 5 and 6, it was agency (but not experience) that fully mediated the negative effect of erection salience on intentions to hire the target as a security guard or a nurse, respectively. Specifically, a discernible erection decreased Tomasz’s perceived agency, which in turn was a positive predictor of the respondents’ readiness to employ him in either of the two positions.^6^

Additional analyses revealed that the mediation effects of erection salience on hiring intentions were moderated by gender. While the negative effects of overt erection were explained by the decrease in perceived agency among female participants, the mediation effects did not occur for male participants (see Supplemental Material). Thus, it seems that male participants preferred not to hire a target with a visible erection for different reasons than female participants.

## Experiment 3

Experiment 2 showed that erection salience diminished the willingness to hire a target individual and that, at least among female perceivers, this effect was explained by the decrease in perceived agency. However, an unambiguous causal order where erection salience lowers agency, and agency in turn increases hiring intentions, was not established.^7^ We addressed this limitation in Experiment 3 by following an experimental-causal-chain design, which involved the manipulation of both independent and mediating variables, and warrants strong causality inferences (Spencer, Zanna, & Fong, 2005). Because Experiments 1 and 2 demonstrated that the independent variable (erection salience) influences both the proposed mediator (agency) and the dependent variable (hiring intentions), in Experiment 3 only agency was manipulated.

## Method

### Participants

A total of *N *= 203 Poles aged 18–75 years (98 females and 105 males, *M*_*age*_ = 40.55, SD_*age*_ = 14.39) recruited via Ariadna research panel participated in the study online. The minimal sample size was determined to be at least 200 to detect even small effects (*ƒ* = .14) with 80% power and assuming a correlation of *r* = .30 (based on the results of Experiment 2) between the repeated hiring measures. Informed consent was obtained from all individual participants. No observations were excluded.

## Materials and Procedure

This two-group experiment was introduced as research on the hiring process. Following a random assignment to conditions, the participants read vignettes presenting Tomasz, a 33-year-old man looking for a job. Whereas in the control condition only basic information was delivered (i.e., place of residence, family and hobbies), in the high-agency condition Tomasz’s agency-related behaviors (e.g., planning a day ahead, avoiding alcohol and candy consumption) were additionally described. Next, the participants were asked to complete measures of mind perception (manipulation check) and hiring intentions. Perceived agency (*α* = .94, *M* = 5.37, SD = 1.36) and experience (*α* = .89, *M* = 5.01, SD = 1.29) were assessed in exactly the same way as in Experiment 2. Also, the measure of hiring intentions was the same as in Experiment 2. However, this time we assessed hiring intentions in relation to four professions: a security guard (*α* = .94, *M* = 4.48, SD = 1.57), a nurse (*α* = .95, *M* = 4.43, SD = 1.59), an architect (*α* = .97, *M* = 4.21, SD = 1.67) and an accountant (*α* = .97, *M* = 4.34, SD = 1.69). The four professions were presented in a counterbalanced order.

## Results

### Manipulation Check: Agency and Experience Ratings

To check whether our agency manipulation was effective, we performed a 2 (Agency Manipulation: Control vs. High) × 2 (Mental Capacity: Agency vs. Experience) mixed-design ANOVA. While the main effect of the manipulation was not significant, *F*(1, 201) = 0.57, *p* > .250, *η*_*p*_^2^ = .00, we found a main effect of mental capacity, *F*(1, 201) = 30.91, *p* < .001, *η*_*p*_^2^ = .13. These effects were qualified by a two-way interaction between agency manipulation and mental capacity, *F*(1, 201) = 29.60, *p* < .001, *η*_*p*_^2^ = .13. In comparison with the control group (*M* = 5.10, SD = 1.40), in the high-agency condition (*M* = 5.53, SD = 1.30), the mean rating of target’s agency was higher, *F*(1, 201) = 5.12, *p* = .025, *η*_*p*_^2^ = .03. By contrast, control (*M* = 5.10, SD = 1.42) and high-agency (*M* = 5.01, SD = 1.30) conditions did not differ in terms of target’s perceived experience, *F*(1, 201) = 0.78, *p* > .250, *η*_*p*_^2^ = .00. Thus, our agency manipulation proved efficient.

### Hiring Intentions

A 2 (Agency Manipulation: Control vs. High) × 4 (Profession: Security Guard vs. Nurse vs. Architect vs. Accountant) mixed-design ANOVA revealed a significant main effect of agency manipulation, *F*(1, 201) = 3.94, *p* = .049, *η*_*p*_^2^ = .02. In line with our expectations, participants in the high-agency condition (*M* = 4.53, SD = 1.28) were more willing than participants in the control condition (*M* = 4.17, SD = 1.28) to employ Tomasz for any of the four positions. By contrast, neither the main effect of profession, *F*(2.58, 517.49) = 2.19, *p* = .098, *η*_*p*_^2^ = .01, nor the two-way interaction of agency manipulation and profession *F*(2.58, 517.49) = 1.60, *p* = .194, *η*_*p*_^2^ = .01, reached significance. Thus, the results of Experiment 3 allowed us to establish a causal chain starting from erection salience through perceived agency to hiring intentions.

## Discussion

This research provides evidence that erection salience can influence how the mind of a male target is perceived. Across different domains (art and social media) and diverse cultural contexts (British and Polish), high erection salience decreased targets’ perceived agency, but did not affect their perceived experience. High erection salience had practical consequences: It translated to lower intentions to hire a target. Among female (but not male) participants, this effect was explained by the loss of perceived agency.

To the best of our knowledge, the present research is the first to examine the relation between erection salience and mind perception. While previous studies (e.g., Mautz, Wong, Peters, & Jennions, 2013) revealed that penis size determined a target’s attractiveness, they utilized solely flaccid penis stimuli and did not investigate other dimensions of social perception.

Our findings contribute to the sexual objectification and mind perception studies. Past results allowed for the formulation of three competing predictions for the influence of penile erection on agency and experience attributions. In the present research, the minds of men with overt erection (but not of men with flaccid penises) were perceived similarly to the minds of animals: as high in experience and low in agency capacities (Gray et al., 2007). As such, the effects of erection salience resemble the animalistic type of dehumanization, which denies uniquely human traits (e.g., self-control, civility, refinement, moral sensibility, logic and maturity), but not traits reflective of human nature (e.g., emotional responsiveness; see Haslam, 2006).

At the same time, the current results did not support the other two phenomena discussed in the literature. First, penile erection did not lead to literal objectification, which would involve a simultaneous decrease in agency and experience (Heflick & Goldenberg, 2014). Being a signal of sexual desire (an experience-related aspect of mind), erection seems to prevent perceiving men as emotionless objects. This result is congruent with the claim that literal objectification may be specific to female targets (e.g., Fredrickson & Roberts, 1997; Heflick & Goldenberg, 2014). Second, high erection salience did not lead to mind redistribution (Gray et al., 2011b). The decrease in target’s agency was not accompanied by an increase in experience. The lack of effects on experience may originate from the specific meaning attached to penile erection. As a symbol of power (Friedman, 2001), an erect penis may preclude seeing its owner as a harm-sensitive experiencer (Gray et al., 2011). In summary, the present research suggests that the belief expressed in the literature and conventional wisdom requires some qualifications: In the eye of the beholder, sexual arousal strips men of their agentic, but not their experiential mind.

Current results have practical implications. Whereas past research (Rollero & Tartaglia, 2013) revealed that objectification decreases men’s perceived suitability for certain (i.e., high status or stereotypically feminine) professions, the exact process underlying this effect has not been investigated. The present research adds to this line of inquiry by showing that, at least among female perceivers, this effect may be mediated by lowered agency ascribed to objectified male targets. Furthermore, as suggested by the results of Experiments 2 and 3, strong cues such as high erection salience may lower intentions to hire a target in general, regardless of profession. The current research does not, however, allow us to rule out alternative reasons for lower hiring intentions (e.g., target’s non-normative behavior as exemplified by exercising despite a visible erection).

Although the present research consisted of three experimental studies conducted with the use of different methods (computer-assisted Web interview in Experiment 1 and Experiment 3; paper and pencil method in Experiment 2) and among participants of different nationalities (British in Experiment 1 and Polish in Experiments 2 and 3), it nonetheless has some limitations. First of all, some of the reported effects may seem quite small, which may likely be a consequence of underpowered samples. This is the case, for example, in the analysis of the effect of agency manipulation in Experiment 3. Besides recruiting more participants, it would be possible to increase the effect sizes by using more powerful experimental manipulations (e.g., manipulating erection salience with the use of video stimuli). Second, our research utilized solely self-report measures, which may raise doubts related to social desirability bias or ecological validity of our questionnaires. Thus, future research is also needed to understand the influence of erection salience on men’s perceived agency in more realistic settings, which would ideally use behavioral, rather than self-report indicators. Third, future research should examine whether the pattern of results would be similar among people coming from different cultural and demographic backgrounds. Finally, when it comes to demographic variables, Experiment 2 demonstrated that the effect of erection salience on hiring intentions was mediated via target’s perceived agency. However, this effect was only significant among female participants which may suggest that male participants preferred not to hire a target with a visible erection because of reasons other than his alleged intellectual inferiority (e.g., intrasexual competition; Puts, Bailey, & Reno, 2015). Future research should explore the perception of social norms violation by the target as well as potential moderators and mediators of the described relation between erection salience and hiring intentions (among both males and females).

Adverse consequences faced by the targets of objectification are dire. They include social acceptability of violence and harassment directed at them (Loughnan et al., 2013), a tendency to self-objectify among the targets, as well as internalization of body focus and unrealistic body standards, lowered self-esteem, and in extreme cases depression, eating disorders and other serious psychological difficulties (e.g., Loughnan, Baldissarri, Spaccatini, & Elder, 2017, Loughnan et al., 2010). These negative effects are particularly strong if targets belong to low-status groups (e.g., sexual minorities; see Heimerdinger-Edwards et al., 2011; Rohlinger, 2002, Wiseman, & Moradi, 2010). Therefore, it seems especially important to identify the outcomes and processes related to sexual objectification. The present research was intended to serve this broader goal.

## Electronic Supplementary Material

Below is the link to the electronic supplementary material.

Supplementary material 1 (DOCX 48 kb)

## Footnotes

1 Supplemental Material including stimuli employed in Studies 1-3 may be accessed online (https://osf.io/3v6kq/?view_only=f6b9eb08592549a9b3372ed9bf0b6d0e).

2 In a pilot study (*N* = 136), targets presented in the large-size condition were perceived to have significantly larger penises than targets in the small-size condition, *p* < .001, *d* = − 0.63.

3 In order to conceal the purpose of the study, some of the evaluations pertained to the artistic qualities of the drawings. For a complete list of evaluation dimensions, see Supplemental Material.

4 EFAs and CFAs results for the scales employed in Studies 1-3 are available in Supplemental Material.

5 The experimenters recruited one person more than specified in a preregistration form.

6 Excluding observations with regression residuals larger than three *SDs* from the mean (Stevens, 2002) did not change our conclusions (see Supplemental Material).

7 Alternatively, hiring intentions might precede perceived agency.
