# Supplementary material for: An Experiencer, An Animal or An Object? Erection Salience Decreases Men’s Perceived Agency
Source: Arch Sex Behav. 2020 Sep 7;49(8):2993–3003. doi: 10.1007/s10508-020-01800-0 (PMC7641924; doi:10.1007/s10508-020-01800-0)
Supplement: Supplementary file 1 — Supplementary material 1 (DOCX 48 kb) [file 10508_2020_1800_MOESM1_ESM.docx]

**Supplementary Material**

**Study 1**

Evaluations pertaining to the artistic qualities of the drawings were attractiveness, pleasantness, realism and balance.

As shown by EFA, six items measuring mind perception formed a single factor that accounted for 57.12% of variance. However, as revealed by CFA, a two-factor solution fitted data better than a single-factor model, Δ χ^2^ (1) = 17.44, *p* < .001.

Other measures employed in Study 1 were human uniqueness (HU) and human nature (HN) (Bastian, & Haslam, 2010), warmth, competence, primary and secondary emotions, acceptance and blatant dehumanization. The decrease of HU/secondary emotions and no changes in HN/primary emotions would support H2. However, the factorial structure of the items comprising humanness and emotions scales did not reflect expected distinctions between HU vs. HN and primary vs. secondary emotions. As shown by the CFAs (Table S1), neither 2-, 3-, nor 4-factor solution reflected the structure of humanness and emotion items in Study 1. Therefore, we decided not to analyze these variables.

Controlling for participants’ gender or analyzing data from heterosexual participants only (Table S2, Models 1 and 3), did not change the results presented in the main text. Although specific targets received different ratings of mind (Table S2, Model 2), the effect of erection salience on mind perception did not differ between drawings (Figure S1).

Table S1. The fit of humanness and emotions confirmatory models (Study 1).

| **MODEL** | **FACTORS** | **FIT INDICES** |
| --- | --- | --- |
| Humanness 2 factors | 1. HU (10 items) 2. HN (10 items) | χ^2^ (169) = 1314.33, *p* < .001, CFI = .49, RMSEA = .18 |
| Humanness 3 factors | 1. HU (10 items) 2. HN (10 items) 3. Method factor (10 negative items) | χ^2^ (157) = 678.42, *p* < .001, CFI = .77, RMSEA = .12 |
| Humanness 4 factors | 1. HU1 (5 negative items) 2. HU2 (5 positive items) 3. HN1 (5 negative items) 4. HN2 (5 positive items) | χ^2^ (144) = 365.88, *p* < .001, CFI = .90, RMSEA = .08 |
| Emotions 2 factors | 1. Primary emotions (6 items) 2. Secondary emotions (6 items) | χ^2^ (53) = 866.67, *p* < .001, CFI = .26, RMSEA = .27 |
| Emotions 3 factors | 1. Primary emotions (6 items) 2. Secondary emotions (6 items) 3. Method factor (6 negative items) | χ^2^ (45) = 173.15, *p* < .001, CFI = .88, RMSEA = .11 |
| Emotions 4 factors | 1. Primary emotions 1 (3 negative items) 2. Primary emotions 2 (3 positive items) 3. Secondary emotions 1 (3 negative items) 4. Secondary emotions 2 (3 positive items) | χ^2^ (48) = 190.46, *p* < .001, CFI = .87, RMSEA = .12 |

Table S2. Supplementary mixed-design ANOVAs, Study 1.

|  | MODEL 1  Gender as an additional between-subjects factor | | | | MODEL 2  Drawing as an additional within-subjects factor | | | | MODEL 3  Non-heterosexual participants (*N* = 20) excluded | | | |
| --- | --- | --- | --- | --- | --- | --- | --- | --- | --- | --- | --- | --- |
|  | *F* | *df* | *p* | η^2^_p_ | *F* | *df* | *p* | η^2^_p_ | *F* | *df* | *p* | η^2^_p_ |
| Erection salience | 5.70 | 1, 211 | .018 | .03 | 4.56 | 1, 215 | .034 | .02 | 2.96 | 1, 195 | .087 | .015 |
| Penis size | 0.62 | 1, 211 | .433 | .00 | 0.31 | 1, 215 | .580 | .00 | 0.56 | 1, 195 | .457 | .00 |
| Mind capacity | 51.81 | 1, 211 | .000 | .20 | 57.31 | 1, 215 | .000 | .21 | 60.69 | 1, 195 | .000 | .24 |
| Gender | 2.09 | 1, 211 | .150 | .01 |  |  |  |  |  |  |  |  |
| Drawing |  |  |  |  | 73.05 | 2, 430 | .000 | .25 |  |  |  |  |
| Erection salience × penis size | 2.28 | 1, 211 | .133 | .01 | 1.41 | 1, 215 | .236 | .01 | 1.71 | 1, 195 | .193 | .01 |
| Erection salience × mind capacity | 19.90 | 1, 211 | .000 | .09 | 19.30 | 1, 215 | .000 | .08 | 15.68 | 1, 195 | .000 | .07 |
| Erection salience × gender | 0.93 | 1.211 | .336 | .00 |  |  |  |  |  |  |  |  |
| Erection salience × drawing |  |  |  |  | 1.80 | 2, 430 | .167 | .01 |  |  |  |  |
| Penis size × mind capacity | 0.67 | 1, 211 | .415 | .00 | 0.72 | 1, 215 | .396 | .00 | 1.03 | 1, 195 | .312 | .01 |
| Penis size × gender | 0.25 | 1,211 | .616 | .00 |  |  |  |  |  |  |  |  |
| Penis size × drawing |  |  |  |  | 0.41 | 2, 430 | .661 | .00 |  |  |  |  |
| Mind capacity × gender | 0.50 | 1, 211 | .481 | .00 |  |  |  |  |  |  |  |  |
| Mind capacity × drawing |  |  |  |  | 18.47 | 1.94, 417.64^a^ | .000 | .08 |  |  |  |  |
| Erection salience × penis size × mind capacity | 1.11 | 1, 211 | .294 | .01 | 0.50 | 1, 215 | .479 | .00 | .70 | 1, 195 | .404 | .00 |
| Erection salience × penis size × gender | 0.97 | 1, 211 | .326 | .01 |  |  |  |  |  |  |  |  |
| Erection salience × penis size × drawing |  |  |  |  | 1.61 | 2, 430 | .200 | .01 |  |  |  |  |
| Erection salience × mind capacity × gender | 1.63 | 1, 211 | .203 | .01 |  |  |  |  |  |  |  |  |
| Erection salience × mind capacity × drawing |  |  |  |  | 0.04 | 1.94, 417.64^a^ | .959 | .00 |  |  |  |  |
| Penis size × mind capacity × gender | 0.02 | 1, 211 | .890 | .00 |  |  |  |  |  |  |  |  |
| Penis size × mind capacity × drawing |  |  |  |  | 3.23 | 1.94, 417.64^a^ | .037 | .02 |  |  |  |  |
| Erection salience × penis size × mind capacity × gender | 2.60 | 1, 211 | .108 | .01 |  |  |  |  |  |  |  |  |
| Erection salience × penis size × mind capacity × drawing |  |  |  |  | 1.14 | 1.94, 417.64^a^ | .383 | .00 |  |  |  |  |

*Note*. ^a^Greenhouse-Geisser correction applied.

Figure S1. Mean ratings of agency and experience as the function of drawing and erection salience. Error bars show 95% confidence intervals

**Study 2**

In a pilot study (*N* = 98) 10 items measuring agency and experience formed two separate factors in EFA. the experience factor accounted for 36,56%, and agency factor for 13.09% of variance. The same factorial structure emerged in Study 3 – agency and experience factors accounted for 44.70% and 10.47% of variance, respectively.

Hiring intentions items formed two factors in EFA. While the nurse factor explained 38.85% of variance, bodyguard factor accounted for 10.47% of variance.

Gender did not change the interaction between erection salience and mind capacity or the effect of erection salience on hiring intentions. When mind capacities served as the DV, there were significant effects of erection salience (*p* = .003, η^2^_p_ = .04), mind capacity (*p* < .001, η^2^_p_ = .24), interaction between erection salience and mind capacity (*p* = .001, η^2^_p_ = .05), and interaction between erection salience and gender, *p* = .036, η^2^_p_ = .02. The remaining effects were non-significant, *p*s > .163.

For hiring intentions as the DV, there were the significant main effects of erection salience (*p* = .001, η^2^_p_ = .06), and job (*p* = .001, η^2^_p_ = .51). Other effects did not reach significance, *p*s > .061.

Excluding observations with regression residuals larger than three SDs from the mean did not change the mediation effects on hiring intentions. Erection salience decreased willingness to hire a target as bodyguard / nurse by lowering agency, IE = -0.16, 95% CI [-0.36, -0.02] / IE = -0.43, 95% CI [-0.72, -0.21] but not experience IE = -0.03, 95% CI [-0.15, 0.01] / IE = 0.03, 95% CI [-0.01, 0.15].

Additional analyses showed that the mediation effects of erection salience on intentions to hire a target as a bodyguard/nurse were moderated by gender. The negative effect of erection salience on willingness to hire a target as a bodyguard/nurse was explained by agency for female (IE = -0.31, 95% CI [-0.59, -0.12] / IE = -0.55, 95% CI [-0.91, -0.26]) but not male (IE = -0.08, 95% CI [-0.24, 0.06]) / IE = -0.14, 95% CI [-0.43, 0.12]) participants.

**Study 3**

In EFA agency and experience items formed a single factor that accounted for 65.07% of variance. However, a two-factor solution fitted data better than a one-factor model in CFA, Δ χ^2^ (1) = 61.87, *p* < .001.

Hiring intentions items formed three factors that accounted for 56.25%, 16.25%, and 8.84% of variance, respectively. However, the fit of CFA solution with 4 profession factors (bodyguard, nurse, architect, accountant) was acceptable, χ^2^ (42) = 122.89, *p* < .001, CFI = .98, RMSEA = .10.

Gender did not qualify the effect of agency manipulation on willingness to hire a target, *F* _agency × gender_ (1, 199) = 1.12, *p* > .250 , η^2^_p_ = .01, and *F* _agency × job × gender_ (2.57, 511.63) = 0.83, *p* > .250 , η^2^_p_ = .00).

**References**

Bastian, B., & Haslam, N. (2010). Excluded from humanity: The dehumanizing effects of social ostracism. *Journal of Experimental Social Psychology*, *46*(1), 107-113.
